# Proposed Classification System for Submandibular Gland Fossa Anatomy and Its Radiomorphometric Assessment Using Cone-Beam Computed Tomography: A Retrospective Study

**DOI:** 10.7759/cureus.77301

**Published:** 2025-01-11

**Authors:** Sukriti Mukherjee, Atul Anand Bajoria, Sangamesh N. C., S. Bhuvaneshwari, Silpiranjan Mishra, Dhirendra Singh

**Affiliations:** 1 Oral Medicine and Radiology, Kalinga Institute of Dental Sciences, Bhubaneswar, IND; 2 Periodontology, Kalinga Institute of Dental Sciences, Bhubaneswar, IND

**Keywords:** cone-beam computed tomography, dental implants, mandibular canal, salivary glands, submandibular gland fossa

## Abstract

Introduction: The morphology of the alveolar ridges in the posterior mandible has numerous variations that may have fatal complications if not assessed properly. An important aspect in the planning of dental implants is the consideration of the bone morphology and the relationship between the implant and vital anatomic structures such as nerves and blood vessels. The advancements in implantology allow for a streamlined methodology for dental implant placement with high aesthetics and patient acceptance.

Aim: The study aimed to evaluate the depth of the submandibular gland fossa and different morphological variations in the posterior mandible using cone-beam computed tomography (CBCT) and propose a novel classification system for the submandibular fossa depth and concavity angulation.

Methodology: From May 2023 to July 2024, in the Department of Oral Medicine and Radiology at Kalinga Institute of Dental Sciences, Bhubaneswar, a retrospective survey was conducted. The main study analyzed 100 CBCT scans of patients aged 18 to over 51 years, with equal gender distribution. For each scan, a cross-sectional image was obtained, and the inferior alveolar nerve (IAN) was identified. Key measurements included the buccolingual width and depth of the sublingual concavity.

Results: A total of 50 male and 50 female CBCT scans were assessed. The frequency of individuals in each age group was relatively similar, with counts ranging from 24 to 26. The concavity angle for male patients was found to be approximately 61.70±16.230 for the right side, whereas female patients had a mean value of 52.33±8.944 for the right side. Additionally, for the right side, male patients exhibited a mean concavity depth of 1.99±1.165, while female patients had a mean of 1.97±0.688. For the left side, male patients had a mean concavity depth of 1.99±0.958, whereas female patients exhibited a lower mean of 1.40±0.766.

Conclusion: Gender-wise distribution of the study parameters showed that male individuals generally had higher values of concavity depth and submandibular gland fossa than female individuals. Variations of the angle showed a notable difference between genders on the right side.

## Introduction

Tooth loss can occur as a result of many conditions including caries, periodontal disease, trauma, and non-restorable teeth [1]. The treatment options for partially edentulous patients with missing single or multiple teeth are many, for example, it can be a provisional removable partial denture, definitive cast partial denture, resin-bonded prosthesis, fixed partial denture, osseointegrated prosthesis, or implant [2]. Various assessments like osteometry, diagnostic casts, and palpation of the ridges are done before implant placement to assess the anatomical areas of various regions, but this cannot be used for the assessment of the posterior mandible [3]. In the modern practice of dentistry, the use of implants has become integral to the goals of restoring patients to complete form and function [4]. An important aspect in the planning of dental implants is the consideration of the bone morphology and the relationship between the implant and vital anatomic structures such as nerves and blood vessels. The advancements in implantology allow for a streamlined methodology for dental implant placement with high aesthetics and patient acceptance [5]. However, to ensure the success of dental implants, proper diagnosis and treatment planning are needed along with a thorough assessment of the region where the implant is to be placed [6]. The assessment includes the height and width of the available ridge, bone density and quality, position of the dental prosthesis, presence of undercut, and bone depth. Moreover, careful assessment of the related anatomical structures such as the nasal floor, maxillary sinus, incisive nerve, mental foramen, and inferior alveolar nerve is important to avoid any complications [7]. The high resolution and accurate three-dimensional images provided by cone-beam computed tomography (CBCT) make it widely used for implant placement [8]. However, no classification based on concavity depth and angulation has been proposed previously and hence there was felt a need for CBCT-based classification.

## Materials and methods

The present retrospective study was conducted in the Department of Oral Medicine and Radiology at Kalinga Institute of Dental Sciences, Bhubaneswar, India. The ethical committee clearance was obtained from the Institutional Ethics Committee (Ref. No. KIIT/KIMS/IEC/1292/2023) of Kalinga Institute of Medical Sciences, Bhubaneswar, prior to the commencement of the study.

The sample size was calculated using G^*^Power software (Heinrich-Heine-Universität Düsseldorf, Düsseldorf, Germany). The sample size needed to detect an error probability of 0.05 and study power of 0.95 was calculated to be 96 samples with an effect size of 0.35. This figure was rounded to 100 to include an equal number of male (50) and female (50) subject scans.

Sample size selection 

The CBCT scans from May 2023 to July 2024 were selected from the Department of Oral Medicine and Radiology at Kalinga Institute of Dental Sciences, Bhubaneswar. Approximately 150 scans were taken randomly and then according to the selection criteria of the study 100 scans were selected, and a survey was conducted involving patients aged 18 to over 51 years with equal gender distribution.

The selection criteria included CBCT images wherein both right and left mandibular molars were present and scans/images having optimal clarity and resolution without any artifacts. The scans with odontogenic and non-odontogenic lesions in the posterior mandibular region and scans belonging to patients with any congenital/developmental disorders, showing the presence of abnormal ridge morphology due to trauma, and having dental implants in the mandibular posterior region were excluded from the study.

Armamentarium

A desktop computer equipped with an Intel Core i7 11th Generation processor (2.80 GHz) and integrated iRISXE graphics (Intel Corp., Santa Clara, US) was used to process and analyze the CBCT data. The CBCT dataset for each scan was exported in digital imaging and communication in medicine (DICOM) format to an external hard drive. The datasets were then downloaded to the hard drive of a computer and the required measurements were made.

Procedure

The specific areas of interest were both the right and left mandibular first molar region. For each scan, depending on the presence of the first molar, a cross-sectional image was obtained, and the IAN was identified, with measurements taken 2 mm coronal to the IAN, because the recommended distance between the implant and the IAN is at least 1.5 mm [9].

**Figure 1:**
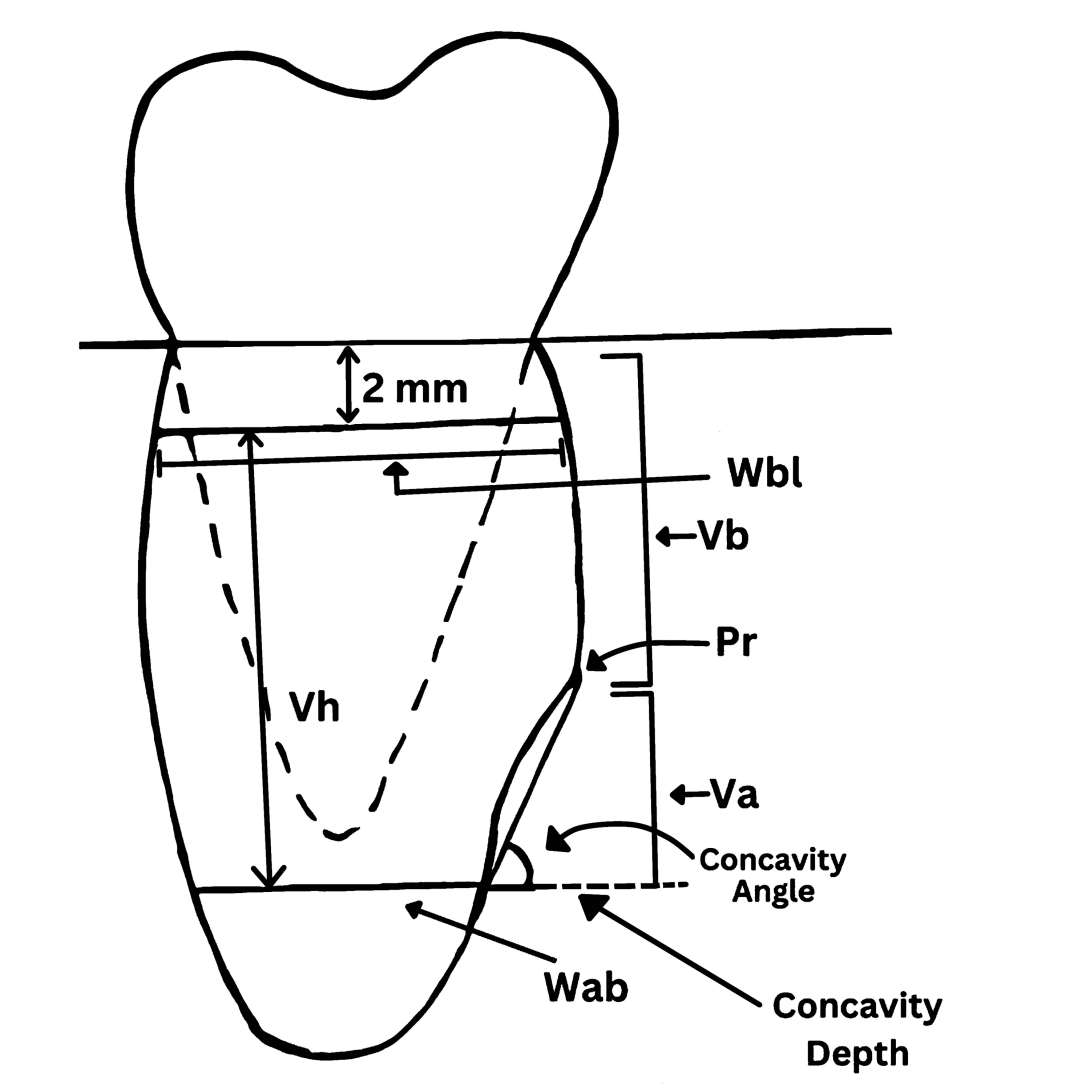
Schematic diagram representing the variables Pr: the most prominent point on the lingual plate; Wbl: buccolingual width that is 2 mm apical to the alveolar ridge crest; Wab: buccolingual width that is 2 mm coronal to inferior alveolar nerve; Vh: height from Wbl to Wab; Va: distance from point Pr to the inferior border of the mandible; Vb: distance from the crest of the alveolar ridge to point Pr The figure was created by the authors.

**Figure 2:**
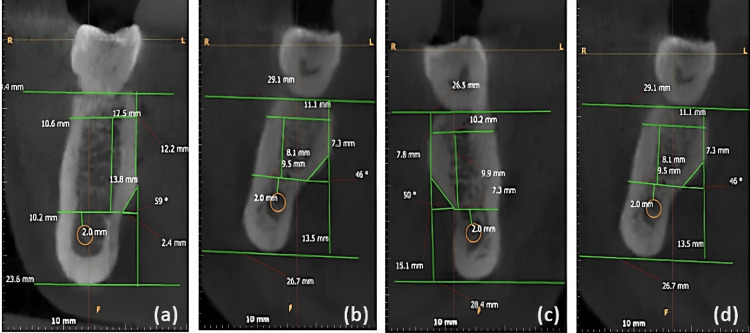
Cross-sectional views of the submandibular gland fossa (a) Male subject, right side; (b) Female subject, right side; (c) Male subject, left side; (d) Female subject, left side

The most prominent point on the lingual plate was recognized (point Pr). The buccolingual width, which is 2 mm apical to the alveolar ridge crest (Wbl) and 2 mm coronal to IAN (Wab), was measured. Three lines were drawn and measured from Wab to 2 mm coronal to IAN (Vh), from point Pr to the inferior border of the mandible (Va), and from the crest of the alveolar ridge to point Pr (Vb). The depth of the sublingual concavity was represented by the angle formed by the intersection of point Pr, Wab, and the horizontal line formed from point A and the intersection with line Va, as illustrated in Figure 1. The submandibular gland fossa was evaluated in cross-sections at 1 mm intervals with 1 mm thicknesses and the deepest regions of the fossa were determined, as shown in Figure 2.

Additional information was collected including age, gender, side, concavity angle, and concavity depth. Data were entered into Microsoft Excel 2016 (Microsoft Corp., Redmond, US) (see Table 11 in Appendices) and analyzed using IBM SPSS Statistics version 25 (IBM Corp., Armonk, US), with categorical variables presented as frequency and percentages, descriptive statistics as means and standard deviations, and one-way analysis of variation (ANOVA) for inferential statistics.

## Results

Demographic characteristics of the study population

Gender Distribution of the Study Population

The study population consisted of 100 individuals in total, evenly divided between male and female genders. Specifically, there were 50 male patients, making up 50% of the population, and an equal number of female patients, also comprising 50% of the population. This balanced distribution ensured equal representation of both genders within the study sample (Table 1).

**Table 1:** Gender distribution of the study population

Gender	Frequency (n)	Percentage (%)
Male	50	50.0
Female	50	50.0
Total	100	100.0

Age Distribution of the Study Population

The frequency of individuals in each age group was relatively similar, with counts ranging from 24 to 26. This indicated a nearly uniform distribution across the age categories. The percentage of individuals in each age group was approximately 25%, showing an even spread. Specifically, the 18 to 30 years group represented 24.0%, the 31 to 40 years group accounted for 25.0%, the 41 to 50 years group comprised 26.0%, and the >51 years group made up 25.0%. The same has been represented in Table 2. 

**Table 2:** Age distribution of the study population

Age	Frequency (n)	Percentage (%)
18 to 30 years	24	24.0
31 to 40 years	25	25.0
41 to 50 years	26	26.0
>51 years	25	25.0
Total	100	100.0

Gender and age-wise distribution of the study parameters based on the side

The gender-wise distribution of study parameters was analyzed for both the right and left sides. On the right side, no significant differences were found between male and female patient scans across all parameters. Specifically, for Wbl, male patients had a mean of 11.60±2.548 compared to female patients, who had a mean of 10.65±1.251, resulting in a p-value of 0.094. For Wab, male patients had a mean of 11.48±2.142 and female patients had a mean of 11.24±1.413, with a p-value of 0.649. For Vh, male patients had a mean of 12.56±2.333 and female patients had a mean of 12.11±1.585, with a p-value of 0.415. For Vb, male patients had a mean of 13.79±2.825 and female patients had a mean of 12.80±2.484, with a p-value of 0.194. For Va, male patients had a mean of 12.94±1.915 and female patients had a mean of 12.08±2.388, with a p-value of 0.172.

On the left side, significant differences were observed for three parameters. For Vh, male patients had a mean of 13.30±1.938 compared to female patients, who had a mean of 11.82±1.327, resulting in a p-value of 0.003, indicating a statistically significant difference. For Vc, male patients had a mean of 14.42±2.162 and female patients had a mean of 12.61±2.35, with a p-value of 0.007, also indicating a significant difference. For Vb, male patients had a mean of 13.08±1.969 and female patients had a mean of 11.91±1.841, with a p-value of 0.035, indicating a significant difference. No significant differences were found for Wc and Wab on the left side, with p-values of 0.916 and 0.390, respectively. These results suggested that while most parameters did not differ significantly between genders, certain parameters on the left side showed statistically significant differences, with male patients generally having higher values than female patients.

The study analyzed several parameters across different age categories and sides (right and left). For parameter Wc, mean values ranged from 10.68 to 11.69 for the left side across age categories, with corresponding standard deviations ranging from 1.45 to 1.99. On the right side, mean values ranged from 10.76 to 11.10, with standard deviations ranging from 1.22 to 3.20. The parameter Wab showed mean values ranging from 10.71 to 11.95 for the left side, with standard deviations ranging from 1.20 to 2.29. On the right side, mean values ranged from 10.82 to 11.76±1.66 to 2.13 across age groups, with standard deviations of 1.66 to 1.86. The parameter Vh exhibited mean values ranging from 12.09 to 13.10 for the left side, with standard deviations ranging from 1.58 to 1.99. On the right side, mean values ranged from 11.94 to 13.01 and standard deviations from 1.41 to 2.30 across age categories. The parameter Vc demonstrated mean values ranging from 12.20 to 14.47 for the left side, with standard deviations ranging from 1.62 to 2.64. On the right side, mean values ranged from 12.75 to 13.81 and standard deviations from 2.26 to 3.10 across age groups. Lastly, the parameter Vb displayed mean values ranging from 12.01 to 13.05 for the left side, with standard deviations ranging from 1.77 to 2.04. On the right side, mean values ranged from 12.08 to 13.32 and standard deviations from 2.20 to 2.42 across age categories. The p-values indicated non-significant differences in means between age categories and sides for most parameters, suggesting relatively consistent distributions across different age groups and sides in the study. The findings of gender and age-wise distribution based on the side are illustrated in Table 3.

**Table 3:** Gender and age-wise distribution of the study parameters based on the side *: statistically significant; Wbl: buccolingual width that is 2 mm apical to the alveolar ridge crest; Wab: buccolingual width that is 2 mm coronal to inferior alveolar nerve; Vh: height from Wbl to Wab; Va: distance from point Pr to the inferior border of the mandible; Vb: distance from the crest of the alveolar ridge to point Pr; Pr: the most prominent point on the lingual plate; N: sample size; SD: standard deviation

	Gender-Wise Distribution									Age-Wise Distribution								
		Right side			P-value	Left side			P-value	Age category (years)	Right side			P-value	Left side			P-value
		N	Mean	SD		N	Mean	SD			N	Mean	SD		N	Mean	SD	
Wbl	Male	23	11.60	2.548	0.094	27	10.67	1.337	0.916	18-30	10	10.7	1.22	0.594	14	10.68	1.99	0.639
										31-40	13	11.69	1.31		12	10.2	1.45	
	Female	27	10.65	1.251		23	10.72	2.055		41-50	12	10.68	0.87		14	11.07	1.64	
										>51	15	11.1	3.2		10	10.77	1.65	
	Total	50	11.08	1.993		50	10.69	1.687		Total	50	11.08	1.99		50	10.69	1.68	
Wab	Male	23	11.48	2.142	0.649	27	11.03	1.744	0.39	18-30	10	11.42	1.66	0.424	14	11.76	2.29	0.335
	Female	27	11.24	1.413		23	11.51	2.163		31-40	13	11.95	1.86		12	10.82	1.2	
	Total	50	11.35	1.77		50	11.25	1.942		41-50	12	11.31	1.13		14	10.71	1.97	
										>51	15	10.82	2.13		10	11.81	2.02	
										Total	50	11.35	1.77		50	11.25	1.94	
Vh	Male	23	12.56	2.333	0.415	27	13.3	1.938	.003*	18-30	10	12.26	1.78	0.513	14	12.37	1.58	.746
	Female	27	12.11	1.585		23	11.82	1.327		31-40	13	13.01	1.41		12	13.1	1.89	
										41-50	12	12.09	2.17		14	12.65	1.99	
										>51	15	11.94	2.3		10	12.27	1.98	
	Total	50	12.32	1.957		50	12.62	1.827		Total	50	12.32	1.95		50	12.62	1.82	
Vb	Male	23	13.79	2.825	0.194	27	14.42	2.162	.007*	18-30	10	13.81	2.26	0.339	14	12.75	2.64	0.228
	Female	27	12.8	2.484		23	12.61	2.351		31-40	13	13.68	2.07		12	14.47	1.62	
										41-50	12	13.67	2.88		14	14.06	2.64	
										>51	15	12.2	3.1		10	13.06	2.3	
	Total	50	13.26	2.665		50	13.59	2.407		Total	50	13.26	2.66		50	13.59	2.4	
Va	Male	23	12.94	1.915	0.172	27	13.08	1.969	.035*	18-30	10	12.19	2.22	0.379	14	12.01	2.09	0.591
	Female	27	12.08	2.388		23	11.91	1.841		31-40	13	12.1	1.84		12	12.47	2.02	
	Total	50	12.48	2.204		50	12.54	1.982		41-50	12	12.08	2.42		14	13.05	2.04	
										>51	15	13.32	2.28		10	12.68	1.77	
										Total	50	12.48	2.2		50	12.54	1.98	

Gender and age-wise distribution of the study parameters irrespective of the side

For the Wc (measurement 1), the mean for male patients was 11.09±2.02, while female patients had a mean of 10.68±1.65. The F-score was 1.258 with a p-value of 0.265, indicating no significant difference between the genders. Wab (measurement 2) showed male patients with a mean of 11.23±1.93 and female patients with a mean of 11.37±1.78. The F-score was 0.125 and the p-value was 0.725, suggesting no significant difference.

For Vh (measurement 3), male patients had a mean of 12.96±2.13 and female patients had a mean of 11.98±1.46. The F-score was 7.229 with a p-value of 0.008, indicating a significant difference between the genders. Similarly, Vc (measurement 4) showed male patients with a mean of 14.13±2.48 and female patients with a mean of 12.72±2.40, with an F-score of 8.401 and a p-value of 0.005, also indicating a significant difference.

For Vb (measurement 5), the mean for male patients was 13.02±1.92, while for female patients, the mean was 12.00±2.13. The F-score was 6.243 with a p-value of 0.014, indicating a significant gender difference. In contrast, angle (measurement 6) had a mean of 58.02±15.66 for male patients and 54.28±13.21 for female patients, with an F-score of 1.666 and a p-value of 0.200, indicating no significant difference.

Lastly, for concavity depth (measurement 7), male patients had a mean of 1.99±1.04 and female patients had a mean of 1.71±0.77. The F-score was 2.350 with a p-value of 0.128, suggesting no significant difference between the genders. Overall, significant differences were found in measurements Vh, Vc, and Vb, while no significant differences were observed in Wc, Wab, angle, and concavity depth. The same has been represented in Table 4.

**Table 4:** Gender and age-wise distribution of the study parameters irrespective of side The table presents age-specific distributions for several study parameters: Wc, Wab, Vh, Vc, Vb, angle, and concavity depth. Each parameter was analyzed across four age categories: 18-30 years, 31-40 years, 41-50 years, and over 51 years. For each age category within a parameter, the table provides key statistical measures. These include N, mean value, SD, and the 95% confidence interval for the mean (lower and upper bounds). Additionally, the table reports the F-score and associated p-value, which assess the statistical significance of differences in means across age groups. The F-score evaluates the variability between age groups relative to variability within age groups, while the p-value indicates whether observed differences in means are likely due to actual differences in the population or could occur by chance. These metrics collectively offer insights into how each parameter varies with age and the reliability of these observations. *: statistically significant; Wbl: buccolingual width that is 2 mm apical to the alveolar ridge crest; Wab: buccolingual width that is 2 mm coronal to inferior alveolar nerve; Vh: height from Wbl to Wab; Va: distance from point Pr to the inferior border of the mandible; Vb: distance from the crest of the alveolar ridge to point Pr; Pr: the most prominent point on the lingual plate; N: sample size; SD: standard deviation

	Gender-Wise Distribution								Age-Wise Distribution							
	Gender	N	Mean	SD	95% confidence interval for mean		F-score	P-value	Age (years)	N	Mean	SD	95% confidence interval for mean		F-score	P-value
													Lower bound	Upper bound		
					Lower bound	Upper bound										
Wbl	Male	50	11.09	2.02	10.52	11.67	1.258	0.265	18-30	24	10.71	1.68	10.01	11.42	0.102	0.959
	Female	50	10.68	1.65	10.21	11.15			31-40	25	10.97	1.55	10.33	11.61		
									41-50	26	10.89	1.33	10.35	11.43		
									>51	25	10.97	2.65	9.87	12.07		
	Total	100	10.89	1.84	10.52	11.25			Total	100	10.89	1.84	10.52	11.25		
Wab	Male	50	11.23	1.93	10.69	11.78	0.125	0.725	18-30	24	11.62	2.02	10.767	12.47	0.52	0.67
									31-40	25	11.41	1.65	10.728	12.09		
	Female	50	11.37	1.78	10.86	11.87			41-50	26	10.99	1.63	10.33	11.65		
	Total	100	11.3	1.84	10.93	11.67			>51	25	11.21	2.1	10.34	12.08		
									Total	100	11.3	1.84	10.93	11.67		
Vh	Male	50	12.96	2.13	12.35	13.57	7.229	.008*	18-30	24	12.32	1.63	11.63	13.01	1.161	0.329
									31-40	25	13.05	1.62	12.38	13.72		
									41-50	26	12.39	2.05	11.56	13.22		
	Female	50	11.98	1.46	11.56	12.39			>51	25	12.11	2.15	11.22	13		
	Total	100	12.47	1.89	12.09	12.84			Total	100	12.47	1.89	12.09	12.84		
Vb	Male	50	14.13	2.48	13.43	14.84	8.401	.005*	18-30	24	13.19	2.5	12.13	14.24	1.947	0.127
	Female	50	12.72	2.4	12.03	13.4			31-40	25	14.06	1.87	13.28	14.83		
	Total	100	13.42	2.53	12.92	13.93			41-50	26	13.88	2.7	12.79	14.97		
									>51	25	12.54	2.78	11.39	13.69		
									Total	100	13.42	2.53	12.92	13.93		
Va	Male	50	13.02	1.92	12.47	13.57	6.243	.014*	18-30	24	12.08	2.1	11.2	12.97	1.041	0.378
									31-40	25	12.28	1.9	11,49	13.06		
	Female	50	12	2.13	11.4	12.61			41-50	26	12.6	2.23	11.7	13.5		
									>51	25	13.06	2.08	12.2	13.92		
	Total	100	12.51	2.08	12.1	12.93			Total	100	12.51	2.08	12.1	12.93		
Angle	Male	50	58.02	15.66	53.57	62.47	1.666	0.2	18-30	24	58.33	18.08	50.7	65.97	1.099	0.354
									31-40	25	52.16	12.43	47.03	57.29		
	Female	50	54.28	13.21	50.53	58.03			41-50	26	58.69	15.02	52.62	64.76		
	Total	100	56.15	14.53	53.27	59.03			>51	25	55.4	11.81	50.52	60.28		
									Total	100	56.15	14.53	53.27	59.03		
Concavity depth	Male	50	1.99	1.04	1.69	2.29	2.35	0.128	18-30	24	1.7	1.06	1.254	2.155	0.602	0.615
									31-40	25	1.86	0.77	1.542	2.178		
	Female	50	1.71	0.77	1.49	1.93			41-50	26	1.79	0.93	1.418	2.175		
									>51	25	2.04	0.93	1.662	2.434		
	Total	100	1.85	0.92	1.66	2.03			Total	100	1.85	0.92	1.669	2.037		

Variation of the concavity depth

Based on Age

Table 5 presents the variation in concavity depth across different age groups. For individuals aged 18 to 30 years, the mean concavity depth was 1.85±1.11. This group showed a non-significant difference in concavity depth across its members, as indicated by a p-value of 0.338. In the 31 to 40 years age bracket, the mean concavity depth slightly increased to 1.97±0.93. However, specific p-values for this and subsequent age groups were not provided, making it challenging to determine statistical significance. Among those aged 41 to 50 years, the mean concavity depth decreased to 1.67±0.92. In contrast, individuals over 51 years old exhibited the highest mean concavity depth at 2.31±0.76. Further analysis across all age groups, totaling 50 observations per group, indicated an overall mean concavity depth of 1.98±0.92. The absence of detailed p-values for age groups beyond 18 to 30 years limited conclusive statements about statistical significance across different age cohorts.

**Table 5:** Variation of the concavity depth based on age N: sample size; SD: standard deviation

	Age	N	Mean	SD	P-value	N	Mean	SD	P-value
Concavity Depth	18 to 30 years	10	1.85	1.11	.338	14	1.60	1.05	.848
	31 to 40 years	13	1.97	.93		12	1.73	.55	
	41 to 50 years	12	1.67	.92		14	1.90	.97	
	>51 years	15	2.31	.76		10	1.65	1.05	
	Total	50	1.98	.92		50	1.72	.91	

Based on Gender

The analysis of concavity depth based on gender revealed noteworthy findings (Table 6). For the right side, male patients (N=23) exhibited a mean concavity depth of 1.99±1.165, while female patients (N=27) had a mean of 1.97±0.688. The overall mean concavity depth for the right side, considering both genders, was 1.98±0.928. The p-value of 0.938 indicated no significant difference between male and female patients for the right side concavity depth. The left side showed a significant difference between genders. Male patients (N=27) had a mean concavity depth of 1.99±0.958, whereas female patients (N=23) exhibited a lower mean of 1.40±0.766. The combined mean for the left side was 1.72±0.915. The p-value of 0.022 signified a statistically significant difference in concavity depth between male and female patients on the left side, suggesting that gender plays a role in the variation observed.

**Table 6:** Variation of the concavity depth based on gender N: sample size; SD: standard deviation

	Gender	Right Side			P-value	Left Side			P-value
		N	Mean	SD		N	Mean	SD	
Concavity Depth	Male	23	1.99	1.165	.938	27	1.99	.958	.022*
	Female	27	1.97	.688		23	1.40	.766	
	Total	50	1.98	.928		50	1.72	.915	

Variation of the angle

Based on Age

Table 7 presents the variation in angles across different age groups. The sample was divided into four age categories: 18 to 30 years, 31 to 40 years, 41 to 50 years, and over 51 years. For the 18 to 30 years age group, the mean angle was 58.90±17.54 with a p-value of 0.421. Similarly, the mean angle for another group within the same age range was 57.93±19.10 with a p-value of 0. 539. In the 31 to 40 years category, the first group's mean angle was 54.23±14.14, while the second group had a mean angle of 49.92±10.43. For the 41 to 50 years age group, the first group's mean angle was 61.25±15.74, and the second group’s mean was 56.50±14.59. In the over 51 years age group, the first group's mean angle was 53.53±6.22, whereas the second group's mean angle was 58.20±17.25. The total mean angle for all age groups combined was 56.64±13.52 in the first dataset and 55.66±15.60 in the second dataset. The analysis indicated variability in the mean angles across different age groups, with standard deviations reflecting the degree of dispersion within each group. 

**Table 7:** Variation of the angle based on age N: sample size; SD: standard deviation

	Age	N	Mean	SD	P-value	N	Mean	SD	P-value
Angle	18 to 30 years	10	58.9	17.54	0.421	14	57.93	19.1	0.539
	31 to 40 years	13	54.23	14.14		12	49.92	10.43	
	41 to 50 years	12	61.25	15.74		14	56.5	14.59	
	>51 years	15	53.53	6.22		10	58.2	17.25	
	Total	50	56.64	13.52		50	55.66	15.6	

Based on Gender

The study examined the mean angles on the right and left sides, comparing male and female patients (Table 8). For the right side, male patients (N=23) had a mean angle of 61.70±16.230, whereas female patients (N=27) had a mean angle of 52.33±8.944. The difference in means between male and female patients on the right side was statistically significant, with a p-value of 0.013, indicating that the variation is unlikely due to random chance, and suggests a true difference in the population. On the left side, male patients (N=27) had a mean value of 56.57±16.85. The p-value for the difference in means on the left side was 0.709, indicating no statistically significant difference between male and female patients for this side. The total mean angles for the right and left sides were 56.64±13.525 and 55.66±15.60, respectively. This suggested that while there was a notable difference between genders on the right side, such a difference was not observed on the left side.

**Table 8:** Variation of the angle based on gender N: sample size; SD: standard deviation

	Gender	Right Side			P-value	Left Side			P-value
		N	Mean	SD		N	Mean	SD	
ANGLE	Male	23	61.7	16.23	.013*	27	54.89	14.74	0.709
	Female	27	52.33	8.944		23	56.57	16.85	
	Total	50	56.64	13.525		50	55.66	15.6	

Classification system

Based on the results acquired in the course of this study, we propose a novel classification for the concavity angle and concavity depth, which is illustrated in Tables 9-10.

**Table 9:** Classification for the concavity angle

Type	Angle of Concavity
Type 1	90 degrees or more than 90 degrees
Type 2	60 degrees - 89 degrees
Type 3	30 degrees - 59 degrees
Type 4	0 degree - 29 degrees

**Table 10:** Classification for the concavity depth

Class	Depth of Concavity
Class A	0-1 mm
Class B	1-2 mm
Class C	2-3 mm
Class D	>3 mm

As per the proposed classification, the following distributions can be made. According to gender, in Type 1, male patients had higher values than female patients. In Type 2, female patients had higher values than male patients. In Type 3, female patients had higher values than males. According to side, in Type 1, the left side had greater values than the right side. In Type 2, the right side had greater values than the left side. In Type 3, the left side had greater values than the right side.

The difference in means, irrespective of gender, on the right side was statistically significant (p-value of 0.013). The p-value for the difference in means on the left side was 0.709, indicating no statistically significant difference between male and female patients on the left side.

According to gender, in Class A, male patients had greater concavity depth. In Class B, female patients had greater concavity depth. In Class C, male patients had greater concavity depth. In Class D, male patients had greater values. According to side, Class A had greater concavity depth on the left side. In Class B, there was greater concavity depth on the right side. Class C had greater concavity depth on the left side and Class D had greater concavity depth on the left side.

Hence, based on this above novel classification, placement of implants can become more planned and precise, avoiding complications such as perforation of the lingual cortical margin, injury to the lingual nerve, injury to the IAN canal, and dehiscence of buccal and lingual cortical plates.

The overall mean concavity depth for the right side, irrespective of gender, indicated no significant difference (p-value of 0.938). The p-value of 0.022 signified a statistically significant difference in concavity depth between male and female patients on the left side.

## Discussion

The floor of the mouth contains vital structures such as the submental and sublingual arteries in the anterior region which if injured, fatal consequences can occur due to perfused bleeding [1]. The posterior region contains the inferior alveolar nerve, lingual nerve, submandibular gland, and lymph nodes. Injury to any of these vital structures, for example, by a lingual plate perforation from dental implant placement can be fatal due to a variety of complications [2]. Careful assessment of these anatomical structures and proper treatment planning prior to implant placement can reduce and/or eliminate such complications.

This study investigated the submandibular gland fossa depth and morphological variations in the posterior mandible using CBCT. The participant distribution was nearly uniform across age groups, ranging from 24% to 26%, allowing for balanced comparisons.

In the present study, a correlation was found between gender and height of the alveolar ridge. The height of the alveolar ridge above Pr showed higher values for males on the left side, and for females, on the right side, whereas the height of the alveolar ridge below the most prominent point showed no correlation. Watanabe et al. [10] took 79 scans to identify the location of the mandibular canal for the planning of dental implantation. The means of height and width ranged from 27.6 to 31.0 mm and from 10.5 to 15.8 mm, respectively. The height in male subjects was significantly greater than that in female subjects, and the width in male subjects was slightly but not significantly greater than that in female subjects. The morphology of the mandible was classified into three types: Type C (round) (59-61%) was the most common in the posterior region followed by Type A (lingual concavity) (36-39%), whereas Type B (buccal concavity) (58-74%) and Type C (17-36%) were the most common types in the anterior region. Salemi et al. [11], who took 164 CBCT scans (of 77 male and 87 female subjects with a mean age of 43.9±12.3 years), and Chan et al. in 2011 [12], who took 103 CBCT scans, found a correlation only in the height of the alveolar ridge below the Pr point. Whereas, Kamburoğlu et al. in 2015 [13], who took 200 CBCT scans, and Herranz-Aparicio et al. [14], who took 151 scans, found no correlation between the height of the alveolar ridge and gender. This can be due to the difference in gender distribution.

The differences in findings can be attributed to the ethnicity of the population being considered, the sample size used in the study, and differences in the linear measurement made by the radiologist while carrying out the study.

CBCT's importance for detailed anatomical assessments, particularly in complex regions like the posterior mandible, has been evaluated. High-resolution images allow accurate measurement of concavity depth, crucial for detecting variations across age groups and gender [5].

The age-wise analysis showed consistent parameter distributions, with no significant differences among age groups, which was concurrently done in the study. Minimal age-related changes in submandibular fossa depth were reported. Mean concavity depths from 1.85±1.11 in the 18-30 years age group to 2.31±0.76 in the >51 years age group, with no significant p-values, were observed [6].

In the present study, a correlation was found between gender and the width of the alveolar bone at the crest, but there was no correlation found at 2 mm above the IAN. Chan et al. in 2011 [12] found similar findings to the present study in both the width of the alveolar bone at the crest and 2 mm above the level of IAN. The ridge was classified into three types: U (undercut), C (convergent), and P (parallel). Their results showed that Type C (round) was the most common (59% to 61%), followed by Type A (36% to 39%). It may be due to the ethnicity of the population. On the other hand, Herranz-Aparicio et al. in 2016 [14] did not find a correlation between the width of the alveolar bone at the crest and gender, while a correlation was found between the width at the base and gender. This may be due to the different classifications used. Furthermore, Salemi et al. in 2018 [11] found a correlation between the width for both at the crest, at 2 mm above the IAN, and gender.

The present study found no correlation between the height of the alveolar bone, the width of the alveolar bone at the crest, and 2 mm above the IAN, and age. This is in agreement with findings in the study by Herranz-Aparicio et al. [14]. 

The present study found a correlation between the concavity depth and gender, with male patients showing greater depth on the left side. This is in agreement with findings by Ramaswamy et al. [15], Herranz-Aparicio et al. [14], and Chaubal et al. [3]. Salemi et al. [11] conducted a cross-divisional study on 164 CBCT scans (77 males and 87 females with a mean age of 43.9±12.3 years). The following information was collected: type of ridge morphology (U: lingual undercut, C: convex, P: parallel), ridge width, ridge height, the angle between the lingual surface and the line drawn above the canal, the distance between the deepest point in the lingual surface and the line drawn perpendicular to the lingual surface, the distance between the most prominent point of the lingual surface and the ridge crest, and the distance between the most prominent point in the lingual surface and the inferior border of the mandible. ANOVA and t-tests were used to compare mean values of CBCT measurements between gender and age groups. Chan et al. in 2011 [12] found no correlation between the concavity depth and gender. This could be due to different gender categorization. All images were acquired with a CBCT machine in the Department of Periodontics and Oral Medicine, School of Dentistry, University of Michigan, by board-certified oral and maxillofacial radiologists between 2005 and 2009. The imaging parameters were set at 120 kVp, 18.66 mA, scan time 20 seconds, resolution 0.4 mm, and a field of view that varied based on the region scanned. In this study, the mandibular first molar edentulous region was the site of interest.

Though the present study found no correlation between the depth, angulation of the concavity, and age, the measurement should be kept in mind to avoid implant surgical complications. This is in agreement with findings by Salemi et al. [11], Chan et al. [12], and Borahan et al. [2]. Herranz-Aparicio et al. [14] found a negative correlation between concavity depth and age, while Chaubal et al. [3] found a negative correlation between age and concavity height.

In summary, CBCT proves effective in evaluating the submandibular gland fossa and reveals significant gender differences on the left side, important for clinical assessments and surgical planning. The study’s methodology, including standardized landmarks and techniques, ensures consistency. Thus, the novel classification system provides valuable information about the concavity depth and angulation, improving clinical decision-making.

Strengths and limitations 

In this study, we have come up with novel classifications for the submandibular gland fossa depth and concavity angle by drawing inferences from the results of the study, which would help the oral-maxillofacial surgeons and diagnosticians to provide precise guides to the placement of implants, thereby avoiding complications. At the time of the study, we did not find any research proposing a classification of this nature, which provides strength to our study.

The sample size of 100 is insufficient to adequately capture the variability and diversity of all relevant variables within the population. A larger sample size would likely provide a more comprehensive representation of the population, reducing the risk of sampling bias and increasing the reliability of the findings. Additionally, the study exclusively included dentulous individuals, which limits its scope and applicability. Including both dentulous and edentulous patients could have provided a broader understanding of the variables and improved the study's relevance to a wider range of patients. Furthermore, reliance on a single institution database introduces potential biases related to the specific characteristics, practices, or patient demographics of that institution, which may not reflect broader or more diverse populations. This limitation reduces the generalizability of the study findings to other settings or populations.

Future studies should aim to include a larger sample size to ensure a more robust analysis and better representation of the target population. This will improve the statistical power and allow for more accurate detection of variability in the data. To expand the study population, both dentulous and edentulous individuals should be included, which can improve the generalizability of the findings. Multi-institutional collaboration should be done to conduct the study across multiple institutions to minimize biases associated with a single institution database. This approach will enhance the diversity of the sample and provide findings that are more applicable to a wider range of settings.

## Conclusions

This study provides valuable insights into the submandibular gland fossa's morphological variations using CBCT imaging, with significant implications for clinical practice and research. As dental and maxillofacial surgery advances, precise anatomical characterization will enhance surgical safety and efficacy, particularly in implantology and reconstructive procedures. CBCT’s high-resolution, three-dimensional imaging offers detailed visualization and accurate measurements, surpassing traditional radiography. Future improvements in imaging software and machine learning may automate and refine these processes, reducing variability and increasing precision. Integrating CBCT with other diagnostic tools like digital intraoral scanners and MRI could further enhance diagnostic accuracy and treatment planning. As CBCT technology evolves, it will be crucial for developing precise, patient-specific approaches in dentistry, which promise better patient outcomes and advance the field of dental medicine.

## Appendices

**Table 11:** Data collection sheet Wbl: buccolingual width that is 2 mm apical to the alveolar ridge crest; Wab: buccolingual width that is 2 mm coronal to inferior alveolar nerve; Vh: height from Wbl to Wab; Va: distance from point Pr to the inferior border of the mandible; Vb: distance from the crest of the alveolar ridge to point Pr; Pr: the most prominent point on the lingual plate

S. No.	Age	Side	Gender	Wbl	Wab	Vh	Vb	Va	Angle	Concavity Depth
1	53	Right	Male	21.8	17.7	8.8	8.3	15.9	61	3.1
2	48	Left	Female	10.7	10.2	11.6	14.2	12.5	49	2.4
3	34	Right	Male	10.6	14.6	13.3	16.7	15.1	90	0
4	19	Left	Female	14.5	15.1	8.1	8.6	17.1	90	0
5	43	Right	Female	10.7	12	14.5	13.3	12.8	73	1.8
6	54	Right	Female	9.3	10.3	10.2	10.6	14	47	1.9
7	27	Left	Female	10.2	9.3	11.4	13	12.7	49	2.3
8	37	Right	Female	10.6	10.2	12.5	12.2	13.8	59	2.4
9	58	Right	Male	11.4	8.8	11.9	9.6	16.4	48	3.2
10	51	Left	Male	11.7	8.8	11.9	11	16.6	55	2.6
11	44	Left	Male	12	10.2	15.3	15.4	15.3	45	3.4
12	37	Right	Female	12.4	11.9	10.9	10.8	12.5	48	2.8
13	42	Left	Female	13.3	11.5	10.2	10.3	14.6	50	1.8
14	35	Right	Female	10.9	12.8	13.6	15.3	10.7	45	1.5
15	28	Right	Male	11.5	7.2	10.4	9.1	14.9	52	3.2
16	37	Right	Female	12.4	15	10.9	12.5	14.6	39	1.5
17	52	Left	Female	11.9	12.7	9.9	11.9	12.8	47	2.3
18	47	Right	Female	11	13.6	10.4	12	11.7	66	1.6
19	54	Right	Female	11.9	10.4	9.1	10.9	15.3	58	1.1
20	38	Left	Female	10.4	8.8	10.6	13.6	14.4	37	1.3
21	28	Right	Male	11.4	12.5	11	13.5	13.8	47	1.8
22	36	Left	Male	12.2	10.4	10.7	11.4	17.1	45	2.8
23	47	Left	Male	13.8	14	16.2	18.7	13	90	0
24	36	Left	Female	13.5	12	12.3	14.3	13.6	73	1.6
25	59	Right	Female	12	9.6	13.5	11.4	15.3	61	2.6
26	42	Right	Female	10.4	10.9	9.6	7.6	15.9	57	1.8
27	54	Left	Male	12	12.8	10.2	14.5	12.3	90	0
28	24	Left	Male	11.2	12.7	12.2	13.5	13.2	30	3.4
29	22	Right	Male	11.5	12.8	9.8	10.6	15	37	3.2
30	28	Left	Female	12.3	10.2	13	12.7	12.2	48	2.6
31	38	Right	Female	11.5	8.3	10.6	9.3	14	43	3.9
32	43	Right	Female	12.2	12	9.9	8.9	16.7	57	2
33	52	Left	Male	11.4	10.1	16.3	13.2	14.8	56	3.1
34	58	Right	Male	11	10.2	13.6	14	14.1	58	2.6
35	42	Left	Male	9.9	10.2	12.4	13.2	15.8	51	2.8
36	56	Right	Male	10.4	12.2	14.6	15.9	11.5	62	1.7
37	52	Left	Male	10.2	13.2	11	13.5	11.5	53	2.6
38	26	Left	Male	12.5	13.5	12.5	15.9	14.8	68	2.7
39	42	Right	Male	12	12.3	12.3	16.9	10.7	90	0
40	46	Left	Male	10.9	14	12.2	15.4	15.1	90	0
41	28	Right	Male	11.9	13.2	11	14.1	14.9	90	0
42	37	Right	Female	12	11.9	13.3	15.8	9.7	41	1.5
43	57	Left	Female	12.7	15.6	12.2	8.1	11.9	90	0
44	46	Right	Male	10.1	9.3	8	13.6	14.5	90	0
45	48	Left	Male	10.4	9.3	10.6	13	14	56	2.2
46	64	Left	Male	12	9.9	12.4	15.1	12.7	42	1.7
47	28	Right	Male	12.5	12	11.5	14.5	11.1	90	0
48	39	Left	Male	11.2	10.2	11.5	14.3	11.9	32	2.8
49	44	Left	Male	10.2	7.3	9.9	7.8	15.1	50	1.5
50	54	Right	Male	11.1	9.5	8.1	7.3	13.5	46	2.3
51	48	Right	Female	10.4	10.6	13	14	10	44	1.8
52	29	Left	Female	9.5	10.4	12.5	13.4	10.4	52	1.6
53	53	Right	Male	9.6	10.14	14.49	15	11.2	56	1.9
54	47	Left	Female	9.2	10.5	11.4	14.6	11	54	1.7
55	66	Left	Female	9.3	12.2	12.8	13.8	12	48	1.3
56	46	Right	Female	10.2	11.5	13.2	15.7	10.7	47	2.6
57	43	Left	Male	11.7	12.6	15.7	16.6	12.9	55	2.8
58	58	Right	Female	8.1	9.6	11.6	12.5	9.6	47	3.9
59	49	Left	Female	13.2	10.6	13.3	15.2	10.2	52	1.6
60	30	Right	Male	9.6	11.2	14.6	14.9	9.8	53	2.4
61	39	Left	Female	9.1	12.4	12	13.6	10.2	48	1.2
62	20	Right	Female	8.7	11.3	12.5	14.2	9.5	53	1.6
63	34	Left	Male	9.5	12.4	15.4	15.5	11.2	56	1.4
64	58	Right	Female	8.5	9.4	12.3	14.8	10.4	48	1.7
65	44	Right	Female	9.2	10.7	12.5	15.8	9.3	51	1.6
66	38	Left	Male	9.5	12.2	15.6	14.2	10.6	54	1.5
67	38	Left	Male	9.4	10.6	15.1	16.7	11.4	56	1.4
68	62	Right	Female	9.2	11.2	12.5	13.2	10.1	44	1.5
69	29	Left	Male	9.4	11	14	15.4	11.2	56	1.3
70	24	Right	Male	9.6	11.3	14.2	16.2	11	54	2.4
71	41	Left	Female	8.1	9.6	12	13.8	9.6	44	1.7
72	19	Left	Male	9.5	10.6	14.7	15	12	48	2.3
73	32	Right	Female	11.6	9.3	13.5	14	10.8	57	1.9
74	46	Left	Male	9.9	12	13.7	15.4	12.9	51	2.1
75	37	Right	Female	12.2	11.8	13.8	14.3	10.1	55	1.3
76	44	Right	Male	10.6	11.2	15.4	16	11.9	58	2.7
77	25	Left	Male	8.7	10.5	14.4	15.3	10	49	1.9
78	28	Left	Female	10.3	11	12.5	13.7	12	50	1.2
79	36	Right	Male	15.4	13	14.7	15.3	11.3	57	2.4
80	24	Left	Female	8	9.4	12	13.1	9	43	1.3
81	39	Right	Male	10.5	12.1	14.6	13.7	12	52	2.8
82	34	Left	Male	9	10.2	13.8	15	11.8	44	1.3
83	22	Left	Female	8.1	9.8	11.5	12.7	9.8	48	1.8
84	30	Right	Female	9.9	11.8	13	15.9	10.4	51	2.1
85	49	Left	Male	11.7	8	12.6	13.3	10.7	54	2.6
86	59	Left	Female	8.4	12.6	15	16.3	11.8	49	1.7
87	43	Right	Male	11.5	10	13.3	15.1	11	56	2.9
88	48	Right	Female	9.9	11.7	13	15.2	9.8	46	1.3
89	34	Left	Male	8.5	10	14.6	13.5	11	53	2.1
90	38	Left	Female	10.4	9.5	11.3	13.9	12	48	1.8
91	55	Right	Male	12	11.9	13.7	15	12.8	54	3.1
92	61	Left	Female	8.1	10.2	12	13.2	10.4	52	1.2
93	22	Right	Male	11	10.9	14.6	15.1	11.5	62	1.8
94	51	Right	Male	9.8	10	15.2	16.9	13.9	56	2.3
95	59	Right	Female	10.5	11.4	9.6	7.6	15.9	57	1.8
96	29	Left	Female	12.7	15.6	12.2	8.1	11.9	90	0
97	33	Right	Female	11.2	12.5	13	14.7	10	46	1.9
98	38	Left	Male	9.7	11.2	14.3	17.7	14.5	53	1.6
99	28	Left	Female	12.7	15.6	12.2	8.1	11.9	90	0
100	38	Right	Female	10.7	12	14.5	13.3	12.8	73	1.8
